# Low-Dose Oral Challenge Test in Pediatric Patients With Peanut Allergy: Tolerance Assessment of a Trace 5 mg Peanut Test After Symptom Induction With a 500 mg Test

**DOI:** 10.7759/cureus.42245

**Published:** 2023-07-21

**Authors:** Kenta Horimukai, Misako Kinoshita, Noriko Takahata

**Affiliations:** 1 Department of Pediatrics, Jikei University Katsushika Medical Center, Tokyo, JPN

**Keywords:** precautionary allergen labelling, peanut hypersensitivity, peanut allergy, oral challenge test, child

## Abstract

Introduction

Peanut allergy (PA) represents a significant public health concern, particularly prevalent in Western countries. Children at high risk for PA may undergo a low-dose oral food challenge (OFC). However, if the result is positive, complete elimination of peanuts from the diet is recommended, and further trace OFC is typically not performed.

Material and methods

This cross-sectional study retrospectively examined the rate of positive peanut OFC with a total peanut load of 5 mg in children who tested positive with a total peanut load of 500 mg. Patient information was gathered from medical records. The primary endpoint was the rate at which children who tested positive in the OFC with 500 mg of peanut butter also tested positive with 5 mg of peanut butter equivalent.

Results

Among 32 children who underwent an OFC with a total peanut load of 500 mg, two were excluded for not meeting the criteria. Among the remaining 30 children, 14 (46.7%) had a positive 500 mg peanut OFC test, and three (10%) experienced an anaphylactic reaction. Those who tested positive for the OFC had higher peanut-specific and Ara h2-specific immunoglobulin E (IgE) antibodies. An OFC with 5 mg of peanuts performed on 10 of the 14 patients who tested positive for 500 mg of peanuts showed no positive results.

Conclusion

The results of this study suggest that children with severe PA who exhibit positive symptoms to a total peanut load of 500 mg can tolerate a 5 mg dose of peanuts and should be considered for an OFC.

## Introduction

Peanut allergy (PA) is a significant global health issue, with an estimated prevalence of 1%-2% in Western countries [1], and is the leading cause of anaphylaxis [2]. In Japan, the prevalence of PA at 3 years of age is 0.5%, and peanuts remain a frequent cause of food allergies [3]. The Japanese food allergy guidelines recommend that children at high risk of developing PA undergo an oral food challenge (OFC) with a "low dose" of peanuts (0.1-0.5 g) [4]. However, the induction of symptoms during "low-dose" OFC poses a high risk of adverse reactions, leading to hesitation among clinicians and parents to conduct further OFC testing [5]. Consequently, physicians often advise patients to eliminate peanuts from their diet. Although the complete elimination of peanuts from a patient's life may reduce the risk of exposure, it could also result in an irrational fear of peanut presence in the environment [6].

A recent meta-analysis examining peanut OFC tests in children and adults with suspected PA found that 4.5% of participants reacted to 5 mg of peanut protein, leading to anaphylaxis [7]. The findings of the meta-analysis for all participants with suspected PA may increase the perception of risk for the subgroup of individuals who have already tested positive for OFC with small amounts of peanuts and are, therefore, at high risk. Individuals who test positive for "low-dose" peanut OFC may face significant barriers to performing additional OFC because of their increased perception of risk in performing even lower doses of OFC. Thus, further investigation is needed to determine whether individuals who are already suspected of having PA in small amounts can tolerate even smaller amounts of peanut exposure. However, very few trials have undertaken additional trace peanut OFC tests in groups that have already tested positive for small amounts of peanut OFC-those presumed to be in the high-risk category. Consequently, we conducted an OFC using a total peanut load of 5 mg (equivalent to 1.3 mg of peanut protein) in children with known severe PA who had previously tested positive in a peanut OFC with a total peanut load of 500 mg. In other words, this study does not aim to observe symptom induction by incrementally 'increasing' the loading dose. Instead, it aims to 'decrease' the loading dose further and examine the rate of symptom induction in a group where symptoms have already been triggered by a small loading dose. Such a viewpoint has seldom been taken in previous studies.

## Materials and methods

Study design and informed consent

This cross-sectional study retrospectively examined the proportion of children with positive peanut OFC with a total peanut load of 5 mg among those who tested positive for peanut OFC with a total peanut load of 500 mg. The primary physician instructed the parents directly on the preparation method for the 5 mg OFC. The ethical aspects of this study were carefully considered and addressed. The parents/guardians were informed orally and in writing regarding the potential risks of OFC, and written informed consent was obtained. The study protocol was approved by the Ethical Review Committee of our institution (approval number 34-373: 11527). This study adhered to the Ethical Guidelines for Life Sciences and Medical Research Involving Human Subjects in Japan. The principles of the Declaration of Helsinki were followed throughout the study.

Objectives

Between January 1, 2015, and November 30, 2022, our medical center conducted OFCs in pediatric patients aged 16 years or younger who presented with suspected PA. A total peanut-loading dose of 500 mg OFC was administered, and 5 mg OFC was administered to children with symptom induction. In the first phase of OFC, if whole peanuts were used, one peanut (approximately equivalent to 1 g) was split, and half a peanut (approximately 500 mg) was administered as the total challenge dose. Conversely, if peanut butter was used, a quantity weighing 500 mg was used. In the second phase of OFC, a total peanut load of 5 mg OFC was used with peanut butter diluted in cow’s milk butter. We procured patient information from medical records, including age, sex, history of symptom provocation, history of anaphylaxis, current atopic dermatitis, and current bronchial asthma. The inclusion criteria for the study were participants who had completed the above-mentioned two OFCs and had their peanut-specific and Ara h2-specific IgE antibody titres measured in the year before and after the 500 mg total peanut load OFC. Children whose test results and medical history could not be obtained due to inadequate entries in their electronic medical records were excluded. Because peanut butter diluted with cow's milk was used for the OFC, children with known allergies to cow's milk were excluded.

Total immunoglobulin E (IgE) levels and serum-specific IgE antibody titers

Total IgE levels, peanut-specific IgE antibody titers, and Ara h2-specific IgE antibody levels were evaluated using the ImmunoCAP assay system (Thermo Fisher Scientific, Uppsala, Sweden). Specific IgE antibody titers <0.1 UA/mL were recorded as 0.09 UA/mL, whereas those >100 UA/mL were recorded as 101 UA/mL. This study utilized concentrations of peanut-specific and Ara h2-specific IgE antibodies measured within one year before and after the OFC. In this study, no measurements were taken for components other than Ara h2 (Ata h1, Ara h3, Ara h6, Ara h9, etc.).

OFC

For participants <5 years of age, smooth peanut butter (Saniku Foods Co., Ltd., Chiba, Japan), free of salt and sugar, was used to prevent inadvertent aspiration into the respiratory tract. Both roasted and boiled peanuts were permitted for use in the OFC, based on parental preference.

The participants consumed one-quarter of the peanuts every hour, for a total of half of the peanuts (approximately 500 mg). Peanut butter (250 mg) was administered every hour, equivalent to a quarter of the peanut dose, for a total dose of 500 mg. The OFC was conducted in a hospital setting, and participants remained as inpatients for follow-up until the following day. The symptoms in the OFC examination were assessed via grade stratification (ranging from 1 to 5) based on Sampson’s classification [8].

OFC testing with 5 mg of peanut butter was conducted within six months of the initial positive OFC test for patients who tested positive with 500 mg of peanuts. The interval between the two OFC tests was not explicitly defined; however, once the results from the 500 mg peanut OFC test were available, the 5 mg peanut OFC test was promptly scheduled. As a result, the interval between the two OFC procedures was a minimum of one week but did not exceed six months. Patients who were Grade 1 or below on the 500 mg OFC test were administered a single dose of peanut butter equivalent to a total load of 5 mg on an outpatient basis and monitored for a minimum of 2 h. Patients who were Grade 2 or higher on the 500 mg OFC test underwent the OFC test as inpatients. The peanuts were diluted 100-fold from a preparation of 1 g of unsalted and unsweetened peanut butter mixed with 100 g of regular butter, homogenized by heating in hot water, cooled, and solidified. The 100-fold diluted peanut OFC was performed under inpatient supervision. Diluted butter was administered in two divided doses (0.1 and 0.4 g) every hour, and the patients were observed until the next day.

Symptoms were categorized as cutaneous (urticaria, erythema, pruritus), respiratory (cough, wheezing, hoarseness, dyspnea), gastrointestinal (vomiting, diarrhea, prolonged abdominal pain), cardiovascular (hypotension), or neurological (confusion). If the OFC medical practitioner deduced that the patient had a positive result, antihistamines (fexofenadine or epinastine) were administered to patients <7 years of age and loratadine to patients >7 years of age. Additionally, in accordance with the pharmacological agents administered at our medical institution, inhaled beta-adrenergic agonists (salbutamol), corticosteroids (prednisolone), or intramuscular epinephrine in the lateral thigh were administered as necessary. An overseeing medical practitioner ascertained whether the OFC findings were positive or negative, awaiting adjudication or anaphylaxis. Participants who experienced grade 2 or higher triggering symptoms, as well as those clinically diagnosed with anaphylaxis by a doctor, were considered to be anaphylactic cases. Awaiting adjudication refers to cases in which obvious objective clinical symptoms were lacking and positive determination was difficult. The details of the OFC were reviewed and retrospectively obtained from medical records.

Endpoints

The primary endpoint was the rate at which children who tested positive in the OFC with 500 mg of peanut butter also tested positive with 5 mg of peanut butter equivalent. The secondary endpoints included the positivity rate of the 500 mg total peanut load OFC, the sensitivity and specificity of peanut-specific and Ara h 2-specific IgE antibody titers, the area under the curve (AUC), and the incidence of adverse events during the 500 mg and 5 mg total peanut load OFC.

Statistics

Continuous variables are summarized as medians (interquartile range [IQR]), whereas nominal variables are expressed as numbers (percentages). Nominal variables were compared using Fisher's exact test, whereas the Mann-Whitney U test was used to compare continuous variables. Total IgE levels and specific IgE antibody titers were log-transformed using an ordinary logarithm, and statistical significance was defined as p <0.05. In addition, receiver operating characteristic (ROC) curves were generated for peanut-specific and Ara h2-specific IgE antibody titers and model performance was assessed using the AUC. The specificity and sensitivity were computed from the points on the ROC curve closest to the upper-left corner. Statistical analyses were conducted using SPSS Inc. Released 2008. SPSS Statistics for Windows, Version 17.0. Chicago: SPSS Inc., and EZR, Version 1.60 (Saitama Medical Centre, Jichi Medical University, Saitama, Japan), which provides a user-friendly graphical interface for R (The R Foundation).

## Results

Patient characteristics

Most OFC trials used readily available, commercially roasted peanuts. Consequently, it was impossible to examine the differences in allergenicity between boiled and roasted peanuts. OFC was performed in 32 children; however, specific IgE antibody titers were missing in two children within one year before OFC. Therefore, the analysis included 30 cases. The results demonstrated that 14 of the 30 patients (46.8%) experienced positive symptoms during the OFC. Table 1 presents the characteristics of the 30 patients analyzed.

**Table 1:** Characteristics of patients undergoing 500mg OFC with peanuts (N = 30) IgE: immunoglobulin E; IQR: interquartile range.

Characteristics	N (%)
Sex	
Male	22 (73.3%)
Female	8 (26.7%)
Age, median (IQR)	4.9 (3.2–6.7)
History of symptom induction by peanuts, n (%)	11 (36.7%)
History of anaphylaxis due to peanuts, n (%)	4 (13.3%)
Current bronchial asthma, n (%)	10 (33.3%)
Current atopic dermatitis, n (%)	23 (76.7%)
Peanut-specific IgE (UA/mL), median (IQR)	14.1 (5.59–22.1)
Ara h2-specific IgE (UA/mL), median (IQR)	4.3 (0.74–13.0)
Total IgE (IU/mL), median (IQR)	439.5 (135.3–1452.3)

In summary, 36.7% of participants had a history of immediate hypersensitivity to peanuts, 13.3% had a history of anaphylactic reactions, and the median Ara h2-specific IgE antibody titer was as high as 4.3 UA/mL. None of the study participants had undergone a peanut OFC test before taking the 500 mg peanut OFC.

Symptom induction with 500 mg OFC

OFC using a cumulative dose of 500 mg of peanuts yielded a positive reaction in 14 cases, corresponding to 46.7% of the tested individuals. Of these, three (10%) presented with anaphylaxis. The classification of the clinical symptoms is shown in Table 2. None of the patients had clinical symptoms above Grade 4. Four patients (13.3% of the total) were excluded from the OFC results because of inconclusive results owing to mild symptoms such as localized itching in the oral cavity.

**Table 2:** Systematic manifestations of OFC with 500 mg of peanuts (14 OFC-positive cases) OFC: oral food challenge The grade classifications in the OFC results are based on the Sampon classification.

	Grade 1	Grade 2	Grade 3	Grade 4–5	Total
Skin, n	5	6	2	0	13
Gastrointestinal tract, n	3	4	0	0	7
Respiratory tract, n	0	2	2	0	4
Cardiovascular, n	0	0	1	0	1
Neurological, n	0	1	0	0	1

Nine patients received antihistamines, two cases were administered inhaled beta-agonists, four cases received systemic corticosteroids, two cases required inhaled oxygen, and three individuals were administered intramuscular epinephrine injections. Two of the three participants diagnosed with anaphylaxis were administered systemic doses of epinephrine and steroids almost simultaneously as part of the emergency response protocol. One patient whose symptoms rapidly improved following the administration of adrenaline could avoid the systemic administration of steroids. Excluding the four cases for which determination was not possible, the clinical profiles of the positive and negative cases were compared (Table 3).

**Table 3:** Comparison of patient characteristics and laboratory findings in positive versus negative 500 mg peanut food challenge results. IgE: immunoglobulin E; IQR: interquartile range. Age, total IgE, and specific IgE are shown as median values (25–75th percentiles). p <0.05 was considered statistically significant. Fisher's exact test, whereas the Mann-Whitney U test was used to compare continuous variables.

Result of OFC	Positive (N = 14)	Negative (N = 12)	p-value
				Sex			
Male	9 (64.3%)	9 (75%)	0.68
Female	5 (35.7%)	3 (25%)	
Age, median (IQR)	4.0 (3.2–5.6)	5.7 (3.5–8.2)	0.12
History of symptom induction by peanuts, n (%)	3 (21.4%)	7 (58.3%)	0.11
History of anaphylaxis due to peanuts, n (%)	1 (7.1%)	2 (16.7%)	0.58
Current bronchial asthma, n (%)	4 (28.6%)	5 (41.7%)	0.68
Current atopic dermatitis, n (%)	10 (71.4%)	9 (75.0%)	0.60
Peanut-specific IgE (U_A_/mL), median (IQR)	20.2 (11.6–45.7)	13.4 (2.2–19.0)	0.05
Ara h 2-specific IgE (U_A_/mL), median (IQR)	11.8 (4.3–28.9)	0.64 (0.09–11.8)	0.013
Total IgE (IU/mL), median (IQR)	398 (131.3–1452.3)	482.5 (122.5–2899.3)	0.72

Diagnostic performance of specific IgE antibody titers

**Figure 1:**
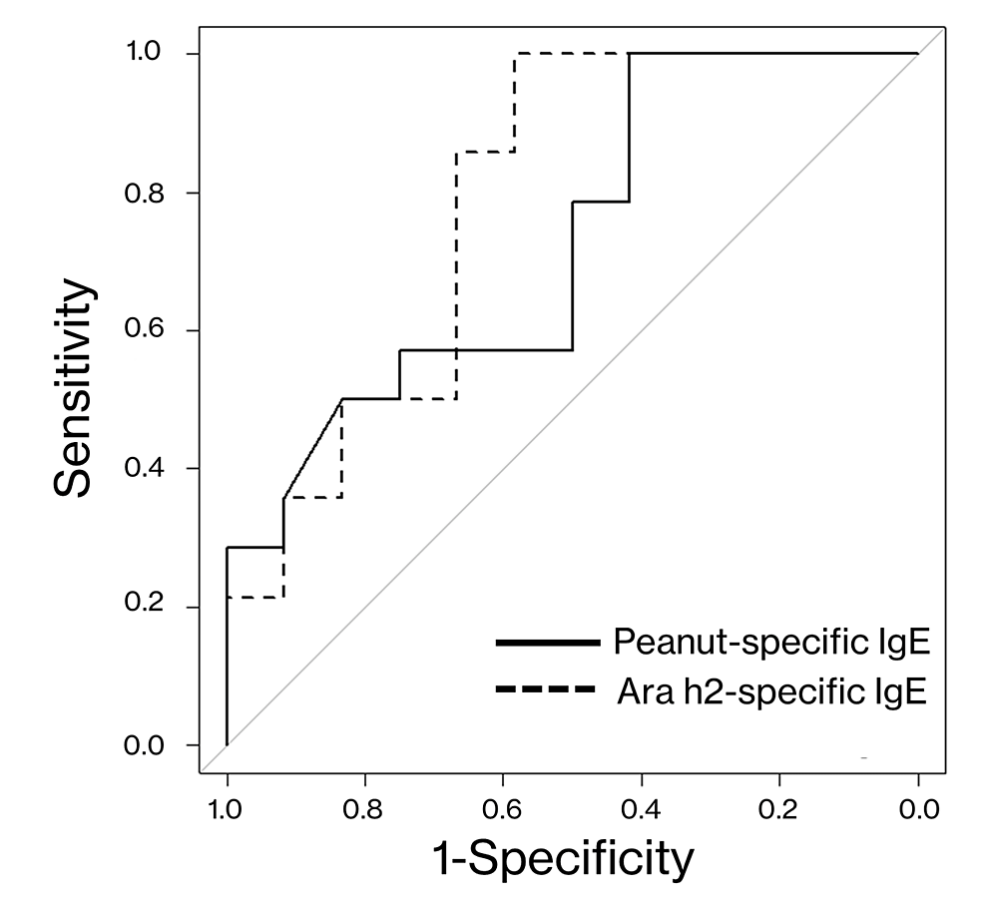
Receiver operating characteristic (ROC) curves for the diagnostic accuracy of severe peanut allergies based on peanut- and Ara h2-specific immunoglobulin E (IgE) antibody levels

Peanut-specific IgE antibody titers were higher, and Ara h2-specific IgE antibody titers were significantly higher in OFC-positive cases. The AUCs for the peanut-specific IgE and Ara h2-specific IgE titers were 0.726 (95% confidence interval, 0.526-0.927) and 0.786 (95% confidence interval, 0.596-0.976), respectively. The optimal cutoff values for the peanut-specific IgE and Ara h2-specific IgE antibody titers were 18.8 UA/mL (sensitivity, 0.75; specificity, 0.57) and 4.27 UA/mL (sensitivity, 0.67; specificity, 0.86), respectively (Figure 1).

OFC with 5 mg total peanut load

A 5 mg peanut OFC was administered to 14 participants with positive 500 mg OFC results to confirm the primary endpoint of this study. All OFCs were performed within six months of the initial 500 mg OFC. Of these 14 patients, 10 underwent a peanut challenge test of 5 mg. Four patients who did not undergo the 5 mg OFC tests were participants whose symptoms elicited by the 500 mg peanut OFC were mild. This included one patient who was Grade 1 but deemed positive by the doctor and three patients who were Grade 2. All three patients who experienced anaphylaxis after the 500 mg peanut OFC tests expressed a desire to proceed with a 5 mg peanut OFC. Consequently, none of the patients showed positive results for the 5 mg peanut OFC tests.

## Discussion

This investigation was carried out in two phases: first, a risk evaluation of a peanut OFC with a total peanut burden of 500 mg, and subsequently, a risk evaluation of an OFC with a total peanut burden of 5 mg for peanut hypersensitivity in patients who exhibited positive results for a total peanut load of 500 mg. In essence, the participants targeted can be presumed to belong to a high-risk group.

The driving force behind this study was the concern of patients with severe PA regarding the presence of trace amounts of peanut allergens in the environment and food [9]. Peanut protein is present in household dust [10], and there is a growing unease that susceptible individuals may experience an allergic reaction from foodborne allergens suspended in ambient air if they are in proximity to an individual consuming a comestible that triggers an allergic response [11]. In addition, various foods may contain trace amounts of peanuts. The packaging of these products is often marked with "Precautionary Allergen Labelling" (PAL) [12].

The guidelines for managing food allergies in Japan recommend a "low-dose" OFC of 0.1-0.5 g of peanuts for children projected to be at high risk. Clearing a total peanut load ranging from 0.1 to 0.5 g (equivalent to 30-150 mg of peanut protein) enhances patient safety [4]. The total "low-dose" OFC load appears reasonable, as research has indicated that the equivalent of 300 mg of peanut protein provides tolerance to 95% of foods containing peanut residue [13]. Nevertheless, in many cases, the amount of peanut protein present in the environment and products that use PAL is lower than the amount of peanut used in "low-dose" OFCs [12]. That is, a difference still exists between the loadings from a "low-dose" OFC and the amount of peanut present in the environment, as well as the amount of peanut protein in PAL products. Therefore, for patients and parents who have experienced symptom induction during "low-dose" OFC, differences in the amount of peanuts in the environment or PAL-formulated products may lead to undue anxiety [14]. Anxiety among caregivers and patients can be deduced from the instance where all three patients who experienced anaphylaxis with a 500 mg peanut OFC subsequently requested a 5 mg peanut OFC.

Greenhawt et al. recently demonstrated the minimal risk associated with environmental exposure to peanuts, such as contact, proximity, and inhalation, for individuals with a tolerance to the equivalent of 1.5 mg of peanut protein [15]. Although it is crucial to acknowledge that trace amounts of allergens may be present in rare cases and cause allergic symptoms even with PAL notation, foods with PAL notation contain either no detectable or very low concentrations of allergenic residues [16]. In other words, it remains essential to determine if an individual with PA can tolerate minimal amounts of peanuts, as this can provide greater comfort to the patient when consuming “possible” foods that contain very low concentrations of allergens. Thus, it is important to further evaluate the risk of trace OFC, even among the presumed higher-risk groups that exhibit symptoms in response to “low-dose” peanut OFC. A future concern necessitates the consideration of the optimal total dosage for children with a high-risk peanut allergy.

Another aspect worth noting is the measurement of specific IgE antibody titres before the OFC. A serum-specific IgE antibody level of ≥4.0 UA/mL targeting Ara h2 is a criterion to avert high-risk OFCs with peanuts [17]. Despite a total peanut load of 500 mg OFC, the median Ara h2-specific IgE antibody titre in the symptomatic group was 4.3 UA/mL, suggesting that it is a valuable indicator. In Japan, only the titer of peanut-specific IgE and Ara h2-specific IgE antibodies can be measured under insurance coverage. Consequently, components other than Ara h2 were not assessed in this study. Therefore, peanut component proteins other than Ara h2 may be useful for differentiation. However, based on previous studies [17], the utility of Ara h2-specific IgE antibody titres seems certain.

In our study, none of the participants who underwent the 5 mg peanut OFC showed adverse reactions, including those with severe PA who had previously experienced induced symptoms during an OFC at a total peanut dosage of 500 mg (with three cases resulting in anaphylaxis). The study participants were expected to represent a cohort with severe symptoms. Therefore, the total peanut load of 5 mg (equivalent to 1.3 mg of peanut protein) used in this study may be tolerated by patients with severe PA, whose symptoms are induced by a total peanut load of 500 mg. These findings support earlier results reported by Greenhawt et al. [15]. Thus, we believe that the peanut loadings used in this study are suitable benchmarks for assessing tolerance to environmental peanut protein exposure.

This study had a few limitations. First, this study had a selection bias due to the attending physician’s decision to perform an OFC with a 500 mg peanut load. However, as mentioned above, the Ara h2-specific IgE antibody titres were high, and the 500 mg total peanut of load OFC induced anaphylaxis in three participants, suggesting that the study population was at high risk. Second, both roasted and boiled peanuts were available as peanut OFC in our study. Despite the different allergenicity between roasted and boiled peanuts [18], in this study, commercially roasted peanuts were used in almost all 500 mg OFCs with peanuts, with the exception of one case where boiled peanuts were utilized. Furthermore, an OFC with a total peanut load of 5 mg was conducted using butter prepared from roasted peanuts, suggesting that these effects were minimal. However, it may be necessary to standardize the food used for OFC in future studies involving high-risk peanut allergy groups to minimize bias. Third, the limited sample size of this study makes it challenging to generalize the findings. Nevertheless, the results of this study align with previous research in several respects and will provide valuable insights for future investigations. Finally, for the 5 mg peanut OFC in our study, roasted peanut butter was diluted with milk butter to allow for the feasibility of testing and ease of weighing. However, the possibility that allergenicity is affected by using milk butter as a solvent cannot be ruled out. Furthermore, mixing peanut butter and water may reduce bias in the results. However, mixing peanut butter and water requires manual skill from the carer, and furthermore, dilutions were made with a mixture of peanut butter and dairy butter to accommodate the taste preferences of participants. In the present study, patients who tolerated peanut butter diluted in cow's milk butter were repeatedly exposed to the same amount during the OFC, and no symptoms were induced. Hence, it is unlikely that the variation in the allergenicity of the diluted peanut butter was significant. However, further investigations are necessary.

Despite these limitations, with the increasing emphasis on PA, research to identify allergic patients who can tolerate less than a "low-dose" total allergen load, including those with severe PA, is important. The ability of patients to tolerate trace amounts of peanuts suggests the potential for oral immunotherapy. For instance, children with food allergies may struggle with the taste of allergenic foods, a significant factor reported as a reason for discontinuing oral immunotherapy [19]. The peanut butter employed in our study contained a higher proportion of dairy products, which effectively diminished the peanut flavor. Therefore, adjusting the ratio of peanut butter to dairy products might enhance the efficacy of oral immunotherapy, and this study encompasses this novel proposition.　

## Conclusions

In conclusion, a significant proportion of pediatric patients with PA may be capable of enduring an OFC in which a total peanut load of 5 mg is administered despite experiencing symptoms manifesting in response to a more sizeable peanut dose of 500 mg. Ara h2-specific IgE antibody titers may prove useful in predicting a positive response to 500mg OFC with peanuts. Our findings also have implications for the starting dose of future oral immunotherapy. However, further investigation into the optimal dosage of OFC for children with more severe peanut allergies is warranted.
